# Editorial Correspondence

**Published:** 1878-07

**Authors:** 


### EDITORIAL CORRESPONDENCE.

Bolingbroke, Monroe Co., Ga., April 30, 1878.

J. G. Westmoreland, M.D.:

*Dear Sir*—I desire to present the case of a lady, described in her own words below, to you for advice. To me it is a novel case. I will close this paper with a short history of her case of deafness.

"It is now over five weeks since my affliction came. I was out in my flower garden having some flowers removed from my pit to the yard. The weather was pleasant. When I came into the house in the afternoon it appeared like every word any one spoke to me was a long way off, or down in a well. Soon after that a song started up in my ear or head, as if some one was playing and singing on some instrument of music. It played several days. Dr. S. proprosed a change of tune, and told me to sing some other tune and see the result. I sang "Hail Columbia." It commenced playing "Hail Columbia," and played it perfectly—beautifully. Since that time it plays a variety of tunes, like I had a music-box in my head—plays the tunes I used to sing long ago. It plays sacred music also. I can stand the music, but sometimes it goes like a river in high water pouring over a dam or precipice. Occasionally it blows like the whistle on a steam engine. This noise upsets me all over. I have had no pain, no fever, and but slight disturbance of my digestion, and am up about my business most of the time. When I awake from sleep it seems to arouse it up rapidly. The roaring seems to cover the sound of the music, but does not obliterate it. I would remark that the tunes change, frequently without my will, though I can change them by singing or playing on the piano."

The lady is a subject of hereditary deafness. Commenced getting deaf about her thirtieth year; she is now 62. She has not heard out of her right ear for 20 years, and this ringing, singing, tuneful noise seems to be in the deaf ear. I have been practicing medicine 48 years, and have not met with a similar case. It works like a phonogram, reviving impressions long since made on the brain.

Please give me your opinion of the case.

Yours truly, D. B. Searcy, M.D.

Monroe County, May 6, 1878.

Dr. J. G. Westmoreland:

*My Dear Sir*—Yours of 2d inst., in answer to mine of the 30th April, was received Saturday, and I thank you for the reply, and shall anxiously await the result of the decision of your society.

In answer to your inquiries: To the first, the subject hears some out of the deaf ear; to the second, there is no uneasiness or pain in either ear but the musical sounds and variations mentioned; to the third question, there is no bubbling—in fact, no air enters the eustachian tube by closing the mouth and nostrils and blowing; to the fourth question I answer, there has been no headache or other unpleasant sensation in the head.

On saturday at dinner she ate a few beans (snaps); at night they soured and passed off by the bowels before morning, leaving some nausea occasionally through the day. During the disturbance of her digestion the noise in the ears and head was worse—more harsh, obliterating for the time, the musical sounds. All mental excitement increases this disturbance, but exercise in walking has a calming effect.

After a tolerably comfortable night's rest she has, this morning, a return of the instrumental music—a waltz, with all the variations, in place of songs. I am confident that this is no illusion. The patient has never had hysterics, never had any earache, no abscess in the internal ear; the drums of each ear perfect; has had occasionally small abscesses form in the external meatus, which discharged spontaneously. Yours truly,

D. B. Searcy.

The novelty of the above case is found in the peculiar musical sounds in the deaf ear. This the author seems to recognize, and desires thought and discussion on the subject. Unnatural roaring, buzzing and screaking sounds are very common in even slight disease of almost any portion of the auditory apparatus, and of course need not be referred to by our venerable and intelligent correspondent. Why it is, however, that memory should transfer the imprint of life's experience in the arrangement of musical sounds to this pathological state, is a matter of deep interest to the neuro-pathologist. The connection with and influence upon the mind had, by physical disease, afford subjects of the highest importance in the study of mental as well as physical disorders. In the above case the mind, without any mental aberration, revives impressions of sounds made upon the auditory aparatus many years ago, and that without any repetition of them by the voice or instrument.

The case is probably one of paralysis of the auditory nerve for the time deafness has existed. If so, may not these musical sounds be the result of partial restoration of this nerve to activity?

The case was reported to the Academy of Medicine, and the opinion of members requested.

Dr Calhoun thought the case one of chronic inflammation of the middle and internal ear, such as is constantly met with, and in which various unnatural sounds prove annoying, and that the mind is in some way connected with the difficulty. He alluded to the treatment of such cases by medicated vapor, liquids, etc.

Dr. Baird thought there existed some mental connection with the diseased ear, by which the seeming sound is perceived by the patient.

